# mSphere of Influence: Communication Is Complicated—Just Ask a Bacterial Cell

**DOI:** 10.1128/mSphere.00580-20

**Published:** 2020-07-08

**Authors:** Paola E. Mera

**Affiliations:** a Department of Microbiology, University of Illinois at Urbana-Champaign, Champaign, Illinois, USA

**Keywords:** *Caulobacter*, cell cycle regulation, molecular communication

## Abstract

Paola Mera works in the field of bacterial developmental biology. In this mSphere of Influence article, she reflects on how the paper “MipZ, a spatial regulator coordinating chromosome segregation with cell division in *Caulobacter*” by Martin Thanbichler and Lucy Shapiro (Cell 126:147–162, 2006, https://doi.org/10.1016/j.cell.2006.05.038) made an impact on her journey discovering the complexities involved in communicative processes that drive molecular mechanisms inside the bacterial cell.

## COMMENTARY

My fascination with communicative processes that drive molecular mechanisms started from my first encounter with an internal conversation between a protein and its substrate and has expanded to an exciting new level—a systems level of communication. A paper that influenced my journey is titled “MipZ, a spatial regulator coordinating chromosome segregation with cell division in *Caulobacter*,” by Martin Thanbichler and Lucy Shapiro (1). This paper is one of those fully comprehensive stories where the authors start where nothing is known and end with pretty much the whole mechanism figured out. Thanbichler and Shapiro identified the mechanism of a key checkpoint in the biphasic cell cycle of Caulobacter crescentus (referred to as *Caulobacter*). This checkpoint is responsible for temporally and spatially coordinating the initiation of chromosome replication, segregation, and cytokinesis. The ingenious communicative processes employed to drive complex mechanisms in bacteria, such as the one revealed by Thanbichler and Shapiro’s story, have become my scientific passion.

My journey through molecular communication began when I was in graduate school in Jorge Escalante-Semerena’s lab. In collaboration with crystallographers and spectroscopists at UW-Madison, we uncovered a fascinating strategy used by adenosyl transferases to facilitate a seemingly impossible intermediate redox reaction. This strategy involved a simple, yet elegant, dynamic communication between the enzyme and its substrate. Using a bulky residue, adenosyl transferases displace the lower ligand of the substrate cobalamin [5-coordinate Co(II) corrinoid], generating a 4-coordinate Co(II) corrinoid that is energetically capable of accepting electrons even from free electron carrier molecules (2–4). Adenosyl transferases also benefit from this conversation in that binding of the substrate liberates highly ordered water molecules from a hydrophobic pocket, thus increasing entropy. As Kiyoung Park (spectroscopist collaborator and since forever friend) would say, the beauty in this system is that the two-directional conversation ultimately results in every player benefitting thermodynamically. The fine mechanistic details uncovered in this project allowed us to appreciate how the Laws of Thermodynamics drive the mechanism of this communicative process. The story from Thanbichler and Shapiro expanded my view of communicative processes to those that include proteins with other proteins, and how the substrates of each of these proteins could influence that upper-level conversation.

Martin Thanbichler and Lucy Shapiro revealed, in remarkable detail, how proteins can communicate to designate place inside the *Caulobacter* cell and specific time over the cell cycle. To designate place, they discovered a measuring stick that came in the form of a protein gradient of a newly identified ATPase protein referred to as MipZ. The question was how *Caulobacter* mediates cytokinesis when it does not encode the two systems (Min and nucleoid occlusion) known to restrict the polymerization of FtsZ (initiator of cytokinesis) to midcell in other species. The authors showed that MipZ inhibits FtsZ polymerization and that this function requires MipZ’s ability to bind and hydrolyze ATP. In actively dividing cells, MipZ was shown to establish a concentration minimum at midcell allowing FtsZ to polymerize and initiate cytokinesis only at midcell. The authors also discovered that MipZ accomplishes this organization by directly interacting with the partitioning protein ParB, which is involved in chromosome segregation following the onset of replication. At the end, this story uncovered how MipZ coordinates this key checkpoint of the cell cycle allowing for the polymerization of FtsZ at the right place and at the right time during the progression of *Caulobacter*’s cell cycle. To my naive mind, this paper exposed the cell as a system that incorporates complex and multidimensional levels of communication.

For my postdoctoral work, I was fortunate to join Lucy Shapiro’s team, the lab that had produced the MipZ paper. There, I became interested in the communicative process responsible for temporally coordinating chromosome replication with segregation. The assumption had been that chromosome segregation was a direct result from chromosome replication. Our data, however, revealed that one could happen in the absence of the other. We observed that chromosome segregation could be triggered independently of replication but, interestingly, was dependent on the replication initiator DnaA (5). As an independent investigator in my lab, I and my colleagues have continued to examine the complexities of this system. We recently found in *Caulobacter* another level of communication between replication and segregation—this time in the opposite direction: a regulator of chromosome segregation coordinating the timing of replication initiation (unpublished work). Here again, we observe that the regulator of chromosome segregation can promote the onset of replication independently of its ability to trigger chromosome segregation. Thus, we now have evidence of a dynamic communication that is engaged in both directions between the regulators that trigger chromosome replication and segregation. An exciting extension of this work is the addition of the environment as another key player in the system. We want to uncover the strategies involved in communicative processes that allow for a system to coexist with its environment. More specifically, my lab is interested in how environmental inputs are fused into the communicative network responsible for orchestrating the progression of the cell cycle. Using a multidisciplinary approach, my goal is to uncover the details of these processes at the molecular and atomic level. That level of understanding will reveal the ingenious strategies of these communicative processes that, aside from driving complex mechanisms inside the bacterial cell, also manage for the whole system to faithfully follow the Laws of Thermodynamics.

Thanbichler and Shapiro’s paper inspired me to venture into the complexities of the multidimensional communicative processes found orchestrating the life of bacterial cells. Beyond this inspiration, however, this paper also highlights that the investigation of communication systems associated with cell cycle regulation is one way that we can begin to more fully understand the bacterial cell from a systemic perspective.
